# Neutrophil Elastase Enhances Sputum Solubilization in Cystic Fibrosis Patients Receiving DNase Therapy

**DOI:** 10.1371/journal.pone.0028526

**Published:** 2011-12-09

**Authors:** Venizelos Papayannopoulos, Doris Staab, Arturo Zychlinsky

**Affiliations:** 1 Department of Cellular Microbiology, Max Planck Institute for Infection Biology, Berlin, Germany; 2 Clinic for Pediatric Pneumology and Immunology, Christiane Herzog Cystic Fibrosis Center, Charité Medical School, Berlin, Germany; University of Tübingen, Germany

## Abstract

Cystic fibrosis patients suffer from chronic lung infection and inflammation due to the secretion of viscous sputum. Sputum viscosity is caused by extracellular DNA, some of which originates from the release of neutrophil extracellular traps (NETs). During NET formation neutrophil elastase (NE) partially processes histones to decondense chromatin. NE is abundant in CF sputum and is thought to contribute to tissue damage. Exogenous nucleases are a palliative treatment in CF as they promote sputum solubilization. We show that in a process reminiscent of NET formation, NE enhances sputum solubilization by cleaving histones to enhance the access of exogenous nucleases to DNA. In addition, we find that in Cf sputum NE is predominantly bound to DNA, which is known to downregulate its proteolytic activity and may restrict host tissue damage. The beneficial role of NE in CF sputum solubilization may have important implications for the development of CF therapies targeting NE.

## Introduction

Cystic fibrosis (CF) is a debilitating hereditary disease that often results in early death of the affected individuals. CF is caused by mutations in the cystic fibrosis transmembrane conductance regulator (CFTR) [1]. The lungs of CF patients produce thick sputum that is difficult to clear and leads to tissue damage by promoting microbial colonization and chronic inflammation. A palliative treatment to reduce sputum viscosity is the administration of recombinant DNase [2], [3], [4], indicating that extracellular DNA contributes significantly to sputum viscosity.

CF sputum contains DNA, neutrophil elastase (NE), myeloperoxidase (MPO) and other neutrophil proteins [5]. Although neutrophil granular proteins are thought to be released through degranulation, the origin of the extracellular DNA is not well established. The extracellular DNA was suspected to originate from necrotic neutrophils and lung tissues rather than infecting bacteria [6]. Recently, CF sputum was found to contain neutrophil extracellular traps (NETs) [7]. NETs are composed of decondensed chromatin in complex with neutrophil antimicrobial proteins (NETs) [8], [9] that are released by dying neutrophils [10] in order to trap and kill microbes [8], [11]. NETs have been implicated in sepsis and in murine pneumococcal pneumonia models [12], [13], [14]. NET release may account for a significant portion of the extracellular DNA and azurophilic granule proteins found in CF sputum.

Neutrophil elastase (NE) is a neutrophil specific protease that is required for NET formation and is implicated in CF pathogenesis. During NET formation, NE processes core histones to promote chromatin decondensation and release [15]. NE is also thought to directly damage tissues in the airways of CF patients [16]. In clinical trials, serum leukocyte protease inhibitor (SLPI) and α1-antitrypsin (A1AT), both NE inhibitors, reduce pulmonary NE activity but are only moderately beneficial to patients [17], [18], [19].

Here, we show that NE degrades the DNA-bound histones in the sputum and enhances the ability of exogenous DNases to reduce sputum viscosity. In addition, we find that all of the NE and myeloperoxidase found in CF sputum are bound to DNA, a key molecular signature of NETs. DNA is known to downregulate the proteolytic activity of NE. Therefore, while NE activity may be damaging to lung tissues, it is also beneficial to patients receiving DNase therapy.

## Results

### NETs are present in cystic fibrosis sputum

**Figure 1:**
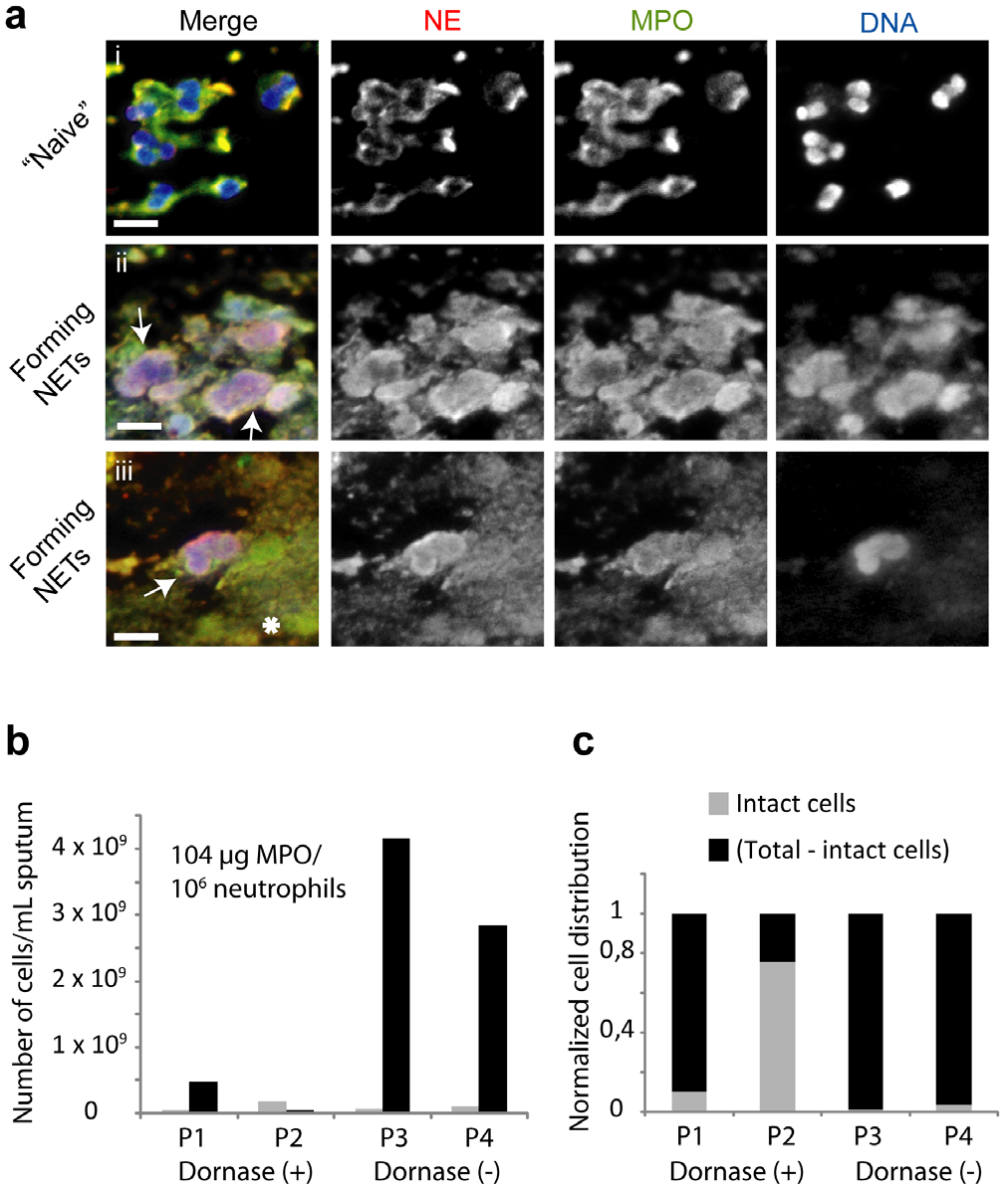
The sputum of cystic fibrosis patients contains neutrophils undergoing NET formation. (a) Representative fluorescence microscopy images of neutrophils undergoing NET formation in the sputum of CF patients. Samples were labeled with antibodies against NE (red) and MPO (green), and with the DNA dye Hoechst (blue). CF sputum contains: (i) intact naïve neutrophils. (ii, iii) neutrophils with decondensed chromatin where NE, MPO and DNA colocalize (arrows), (iii) large aggregates that contain DNA, MPO and NE (asterisk). Scale bar: 10 µm. (b, c) The distribution of intact neutrophils and neutrophils that have made NETs in 2 untreated patients (P1 and P2) and 2 patients receiving DNase therapy (P3 and P4). The estimates are based on a combination of microscopic and biochemical measurements. (b) Intact cells/mL (grey) measured by microscopy, and total cells – intact cells/mL (black). The total number of cells was estimated from MPO activity/mL of sputum measured against a lysate derived from a known number of neutrophils. A value of 104 µg MPO/10^6^ neutrophils was used to estimate the total number of neutrophils (c) The data from (b) plotted as normalized distribution of cells.

We first examined CF sputum derived from 3 CF patients who were not undergoing DNase therapy, for the presence of NETs or neutrophils undergoing NET formation by immunofluorescence microscopy. In addition to intact neutrophils (**Fig. 1, i**) CF sputum contained neutrophils undergoing NET formation (**Fig. 1, ii and iii** **, arrows**). These cells displayed decondensed nuclei that stained with NE and MPO markers. This morphology and the translocation of these azurophilic markers to the nucleus is consistent with the process of NET formation [15], [20]. In addition the sputum contained decondensed extracellular DNA masses that stained positively with NE and MPO antibodies. These amorphous masses resemble NETs that decondensed and fused into large aggregates (**Fig. 1, iii** **, asterisk**).

In order to quantitate the distribution of intact neutrophils and neutrophils that released NETs we counted the number of intact cells in the sputum of 2 untreated patients and 2 patients receiving DNase therapy by microscopy (**Fig. S1**). The total number of neutrophils in the sputum was estimated by comparing the MPO activity in the whole sputum following solubilization with EGTA to the MPO activity of an extract made from a known number of neutrophils. To estimate the number of neutrophils that underwent NET formation we subtracted the number of intact neutrophils from the total number of neutrophils based on MPO activity measurements in whole sputum solubilized in the presence of EGTA (**Fig. 1b and c**). The raw data used for these measurements are presented in **Fig. S2a and b**. Overall, our data indicate a prevalence of NETs over intact neutrophils in CF sputum. Interestingly, the sputum of patients receiving DNase therapy contained significantly lower levels of NET-associated MPO in comparison to the sputum of patients undergoing DNase treatment. In contrast the number of intact cells did not vary dramatically.

**Figure 2:**
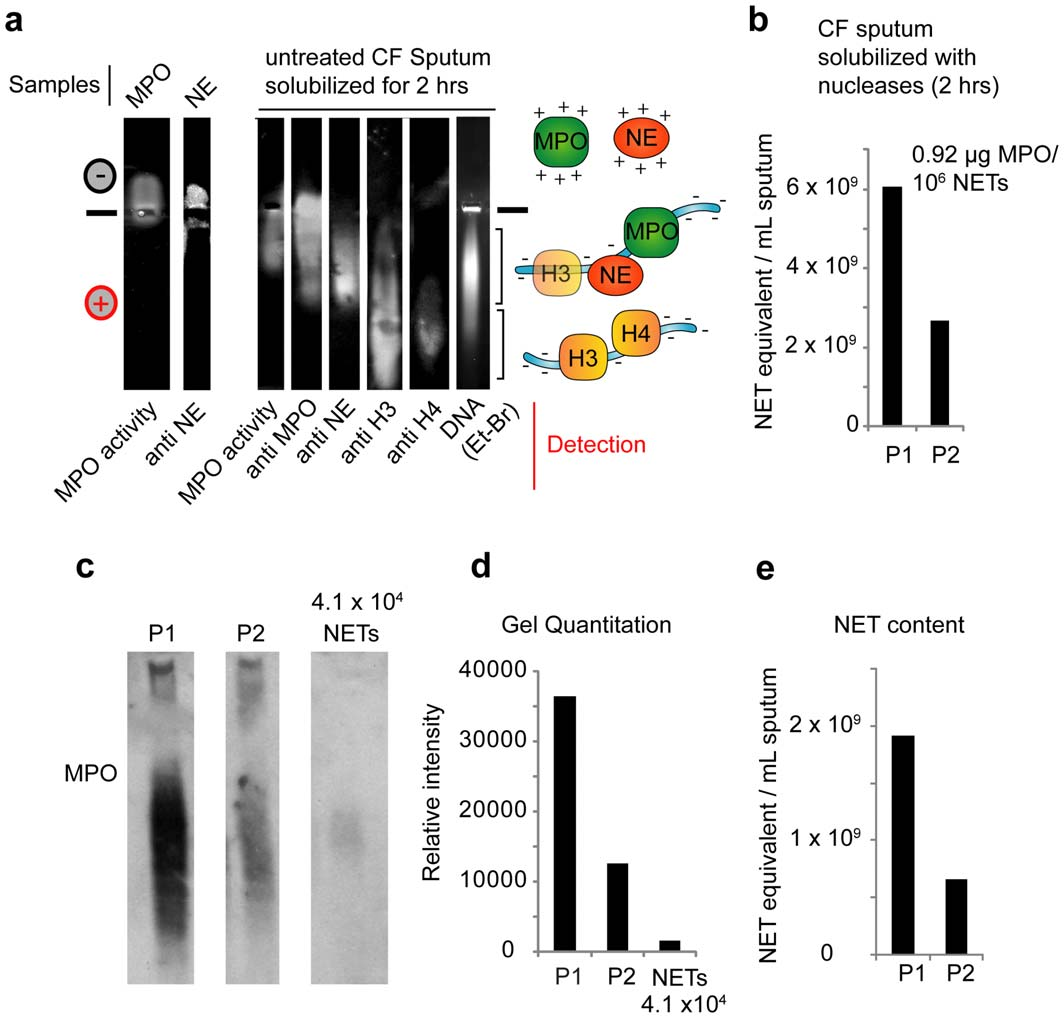
CF sputum contains DNA in complex with MPO, NE and histones. (a) MPO and NE in CF sputum are predominantly bound to chromatin. Purified MPO, purified NE and solubilized sputum supernatant from a representative patient (3–8) digested with nucleases for 6 hrs in the absence of protease inhibitors, resolved by native agarose electrophoresis and blotted to nitrocellulose. MPO was detected by activity prior to gel transfer and by immunoblotting. NE, H3 and H4 were detected by immunoblotting. DNA was detected by ethidium bromide staining. Cartoon representation of the complexes (right) where DNA is depicted in blue and the different proteins are indicated. The cartoons are positioned next to the area of the gel where the corresponding proteins migrate. Similar results were obtained with samples from all CF patients. (b) NET content of CF sputum using the activity of MPO in comparison to the MPO activity of human NETs. The MPO levels in the soluble fraction of CF sputum solubilized with nucleases from 2 patients treated with Dornase were compared to MPO levels in MNase treated NETs from a known number of human neutrophils. Accordingly, 10^6^ NETs treated with MNase gave rise to 0.92 µg soluble MPO. This ratio was used to obtain the NET equivalent/mL in CF sputum samples. (c) Comparison of the MPO levels in CF sputum by detection of MPO in supernatants solubilized with nucleases for 2 hrs, to the MPO content of 4.1×10^4^ NETs, analyzed by native gel electrophoresis and western immunoblotting. (d) Quantitation of MPO levels in (c) using band digitization. (e) NET content in CF sputum based on native gel electrophoresis analysis of sputum supernatants solubilized with nucleases, by comparison of the band intensities in (c). All concentrations refer to the original sputum volume.

A defining characteristic of NETs is the association of chromatin with neutrophil granular proteins such as NE and MPO [8]. We used this property to investigate the presence of NETs biochemically in the sputum of 10 CF patients receiving DNase therapy. To minimize variation due to therapeutic regimes of different patients we further solubilised the sputum with micrococcal nuclease (MNase) in vitro. To detect NET complexes, we resolved solubilized sputum by native agarose gel electrophoresis. In this assay, the highly cationic NE and MPO purified proteins migrate towards the anode (**Fig. 2a**). Interestingly, when the soluble fraction of CF sputum digested for 6 hrs is resolved, NE and MPO co-migrated predominantly with DNA towards the cathode, indicating that these proteins are tightly bound to the highly negatively-charged DNA. Furthermore, an in situ enzymatic assay revealed that the bound MPO is active and therefore folded (**Fig. 2a**).

Histones are also cationic and their migration towards the cathode indicates that they are also tightly bound to the negatively charged DNA in CF sputum. The complexes that contained NE and MPO co-migrated with low amounts of histone H3 and did not contain histone H4 (**Fig. 2a**). This is consistent with previous findings indicating that NE selectively degrades histone H4 in the context of chromatin [15]. It is also likely that CF sputum contains chromatin that originates from necrotic cells and not from NET formation. Consistently, a fraction of histone H3 migrated with DNA that was not bound to MPO or NE (**Fig. 2a**). Alternatively, some of these DNA fragments may also derive from NET material as MPO is thought to be unevenly distributed in the chromatin of NETs [8]. Taken together, these findings suggest that a significant portion of chromatin in CF sputum may originate from NET formation.

To estimate the amount of NET material in the sputum, we compared the MPO activity in solubilized sputum supernatants from two patients receiving DNase therapy, to the activity of MPO derived from a known number of NETs digested with MNase (**Fig. 2b and c**). Interestingly, MNase treated NETs contained only a small fraction of the active MPO found in whole neutrophil extracts (compare **Fig. 1b** and **Fig. 2b**). Furthermore, we quantitated the NET content in the sputum of these patients from the MPO levels detected by native gel electrophoresis and western immunoblotting (**Fig. 2c, d and e**). Our data suggest that CF sputum contains NET products derived from approximately 10^8^–10^9^ neutrophils/mL.

### NE enhances sputum solubilization

**Figure 3:**
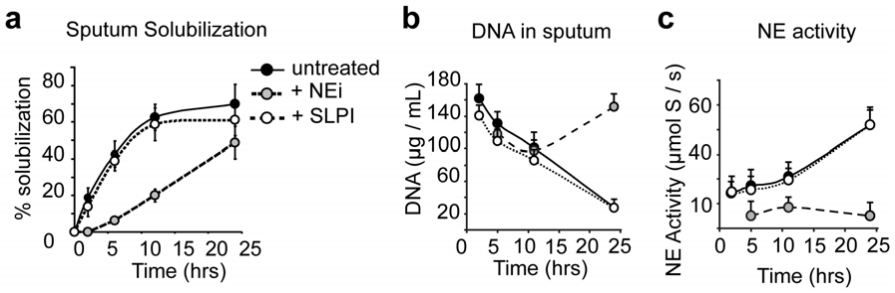
NE enhances nuclease dependent sputum solubilization. Time course analysis of untreated (black circles) and sputum treated with SLPI (open circles), or NEi (grey circles). (a) The sputum was solubilized by DNase administered to patients and MNase. Samples were incubated untreated or in the presence of NEi or SLPI. At the indicated timepoints, the samples were centrifuged at 1000× *g* for 10 min and the soluble volume was measured. The data are plotted as the % of soluble volume against the total volume of the sample and represent the average from individual patient measurements. (b) DNA is degraded in soluble sputum supernatants incubated with nucleases in the absence (black circles) or in the presence of SLPI (open circles) but not when incubated with NEi (grey circles). Soluble aliquots were obtained at the indicated timepoints and DNA was measured with the Quanti-T dsDNA assay. (c) NE activity in solubilized sputum supernatants over time. NE but not SLPI inhibits NE activity in CF sputum. (a, b) Data from a representative CF patient sample. The same trend was obtained in all patients but the overall levels of DNA and NE activity vary significantly amongst samples from different patients.

During NET formation, NE promotes the release of chromatin by processing nuclear histones. We reasoned that NE may affect CF sputum solubility through its ability to modify chromatin density. To investigate the role of NE in sputum solubilization we incubated sputum aliquots with the small molecule NE inhibitor GW311616A (NEi) or SLPI, a serpin protein that inhibits NE. Over the course of the incubation, we centrifuged the samples and measured the volume of soluble supernatant. NEi, but not SLPI, delayed sputum solubilization and supernatants could not be collected before 6 hrs of incubation (**Fig. 3a**).

Since exogenous nucleases promote sputum solubilization by fragmenting the DNA, we examined the role of NE activity in DNA solubilization. We found that DNA digestion by the exogenous nucleases was blocked in samples treated with NEi but not SLPI (**Fig. 3b**). The inability of SLPI to inhibit solubilization is consistent with its inability to effectively inhibit NE activity in CF sputum (**Fig. 3c**). In contrast, NEi inhibited NE activity more efficiently.

**Figure 4:**
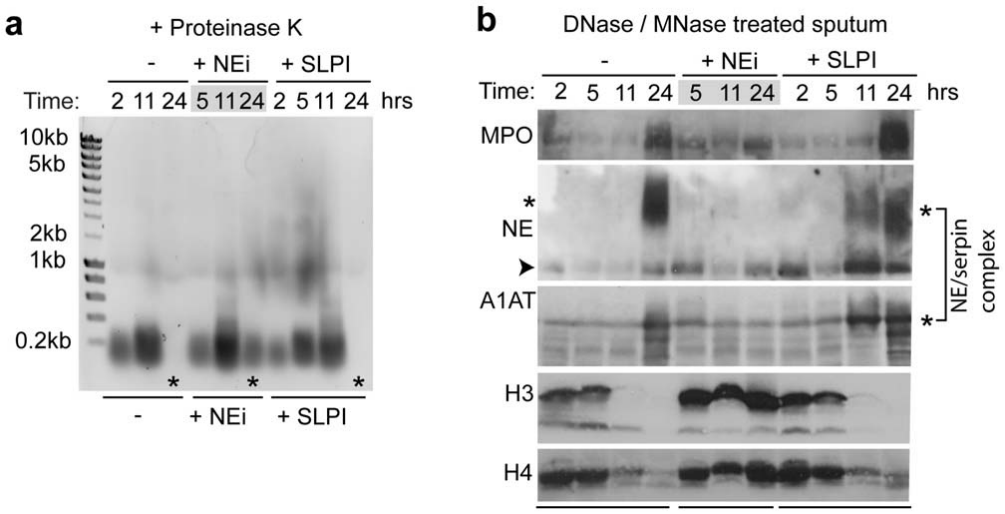
NE degrades histones in CF sputum. (a) The solubilized sputum samples were treated with Proteinase K to digest all proteins and were resolved by agarose gel electrophoresis. DNA was detected by ethidium bromide staining. The released DNA is approximately 200 bp long and is digested over time in samples where NE is active. NEi blocks the digestion of DNA after 24 hrs of incubation (asterisks). (b) MPO, NE and histones, solubilized from nuclease-treated CF sputum in vitro, are degraded over time. Protein degradation depends on the activity of NE. Immunoblot against MPO, histones H3 and H4 in sputum sample supernatants from one representative patient. Sputum samples from each patient in the absence or presence of NEi, or SLPI were resolved by SDS-PAGE electrophoresis. Aliquots were collected at the indicated time points during incubation with nucleases at 37°C. An NE/serpin complex appears (asterisk at approx. 60 kDa) in untreated and SLPI-treated sputum, which is distinct from NE alone (arrowhead). This band is approximately 55 kDa and co-migrates with α-1-anti-trypsin (A1AT) (asterisk). NEi blocks the formation of this higher molecular weight covalent complex between NE and A1AT, indicating that serpins are targeted and deactivated by active NE in CF sputum.

Digestion of proteins in the supernatants with Proteinase K revealed that the solubilized DNA was approximately 200 bp long, corresponding to the size of DNA that wraps around a single nucleosome (**Fig. 4a**).

To investigate whether NE promotes sputum and DNA solubilization by proteolytically processing the histones that package the chromatin we examined the levels of soluble histones over time. In the absence of NEi histones were degraded over time (**Fig. 4b**). In contrast, NEi but not SLPI blocked histone degradation. The inability of SLPI to potently inhibit NE activity in CF sputum is consistent with its reported inactivation via degradation by its target proteases [17], [21]. Consistently, in the absence of NEi, we detected a higher molecular weight moiety of NE that co-migrates with A1AT, a serpin related to SLPI, resulting from the formation of a covalent complex between NE and its inhibitor (**Fig. 4b** asterisks) [22]. This observation suggests that SLPI and other serpins are in fact targeted by proteases in CF sputum and may be overwhelmed by the high concentration of NE. Taken together, these data suggest that NE activity promotes sputum solubilization by degrading histones and increasing the accessibility of the DNA to nucleases.

**Figure 5:**
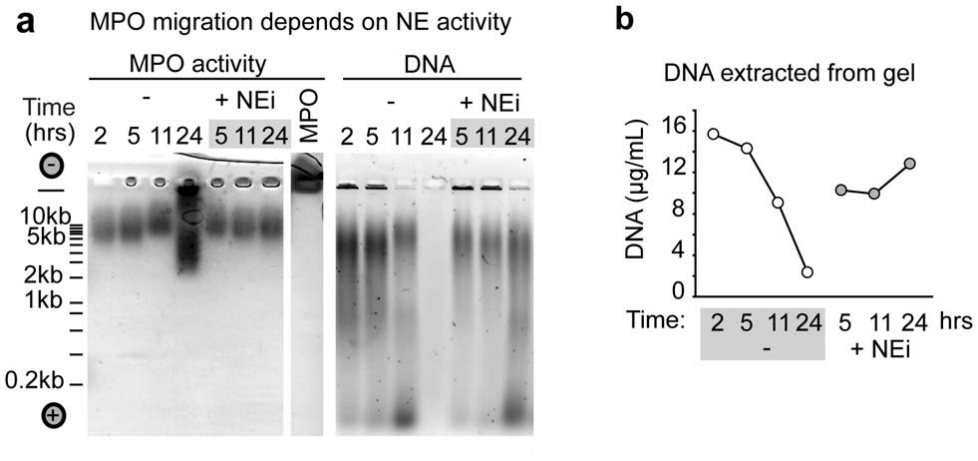
NE, MPO and DNA form a complex in CF sputum. (a) NE-mediated degradation of DNA-bound proteins enhances the degradation of DNA by exogenous nucleases and alters the migration pattern of MPO. Purified MPO or supernatants from sputum samples solubilized with nucleases at the indicated time points, either untreated (−) or treated with NEi (+NEi), were separated by electrophoresis on agarose gels under native conditions. A black circle with a (−) sign denotes the anode, and a red circle with the (+) denotes the cathode during electrophoresis. A line marks where the sample was loaded. MPO was detected by incubating the gel in a solution containing hydrogen peroxide and the MPO substrate O-phenylenediamine, which turns orange upon oxidation by the enzyme (middle panel). DNA was detected by ethidium bromide staining (right panel). The dependence of MPO migration on the presence of DNA and NE activity suggests that these molecules are part of the same macromolecular complex. No soluble sample could be collected at 2 hrs in the presence of NEi. (b) Low levels of DNA can be detected in excised gel slices containing MPO in (a) using the Quant-iT dsDNA assay. (a, b) Data from a representative CF patient sample.

Since NE activity affects the levels of solubilized DNA in CF sputum over time, we hypothesized that if MPO is bound to chromatin we should detect a change in its migration pattern in a native gel that is dependent on NE activity. At 24 hrs of incubation in the absence of NEi, we detected a shift in the MPO migration pattern that is consistent with the near complete fragmentation of DNA molecules. Interestingly, even after near complete nuclease digestion, MPO did not migrate to the cathode, indicating that the protein was still bound to short fragments of DNA (**Fig. 5a**). Using a more sensitive method we detected low levels of DNA in nuclease-treated samples at 24 hrs (**Fig. 5b**) which were undetectable by ethidium bromide staining on native agarose gels (**Fig. 5a**). In contrast, NEi treatment completely blocked the shift in MPO migration pattern (**Fig. 5a**). These results confirm that our native electrophoresis assay detects the association of these positively charged proteins with DNA and can be used for the biochemical detection of NETs in patient samples. Furthermore, the ability of NE activity to influence MPO mobility in this assay suggests that these proteins are part of the same macromolecular complex in CF sputum.

## Discussion

Our findings suggest that a significant amount of extracellular DNA in the sputum of cystic fibrosis patients originates from NET formation. In the past, the extracellular DNA found in CF sputum was thought to originate from dying necrotic or apoptotic neutrophils and epithelial cells [6]. However, in such a case, the chromatin would remain condensed and would not be found in complex with neutrophil granular proteins. NET formation is the only known neutrophil death process that can account for both chromatin decondensation and its association with granular proteins. Our data (**Fig. 2a**) indicate that approximately half of the total amount of DNA in the sputum is complexed with neutrophil granular proteins. The remaining DNA may represent NET fragments that contain low levels of granular markers, or DNA that originates from necrotic cells, independent of NET formation.

We used different approaches to quantitate the NET content in sputum based on the levels of MPO. Notably, the levels of MPO in whole cells and NETs formed by isolated neutrophils in vitro vary significantly and it is unclear which figure should be used as reference. Although here we employed both reference figures, the conditions in the highly dense sputum may enhance the association of MPO during NET formation in contrast NETs derived from isolated neutrophils plated on a dish where MPO is free to diffuse into the supernatant. Despite this problem, our data indicate a strong prevalence of NETs over intact cells in CF sputum. Moreover, while an extended study is required, our limited results suggest that DNase treatment may reduce the overall levels of neutrophil products in sputum. This decrease may arise from solubilization and absorption of cationic proteins prior to sputum clearance, or through degradation due to increased NE activity. Therefore, the NET prevalence in DNase-treated sputum may be understated.

Notably, NE activity enhances chromatin degradation in DNase-treated sputum. Our findings suggest that NE in CF sputum is predominantly associated with DNA (**Fig. 2a**). DNA binding may dampen the deleterious effects of NE on lung tissues since it downregulates the activity of the protease [23]. We and others have found that nuclease treatment increases the presence of NE proteolytic activity in the solubilized sputum [3]. Interestingly, NE appears associated with small fragments of DNA after nuclease digestion, suggesting that administering therapeutic nucleases to patients may not enhance the activity of NE against host lung tissue as dramatically as it would in the complete absence of DNA. In addition to its role in chromatin decondensation, NE has been recently shown to degrade mucin in the airways of CF patients, which is thought to decrease immune defense but could also contribute to sputum solubilization in a chromatin-independent fashion [24].

**Figure 6:**
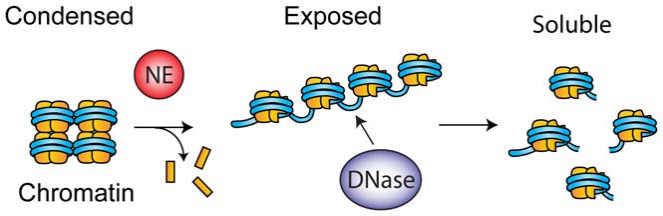
NE synergizes with therapeutic nucleases in promoting CF sputum solubilization. By degrading histones, NE promotes the relaxation of chromatin which exposes DNA to the action of exogenously administered nucleases.

Most importantly, our findings suggest that similar to its function in NET formation, NE promotes chromatin decondensation in CF sputum by proteolytic processing of histones. This processing promotes the decondensation of chromatin which exposes the DNA to digestion by exogenous nucleases (**Fig. 6**). This specific role of NE may have both positive and negative effects for patients. On one hand, NE activity is required for NET formation. Blocking NE activity in CF patients may lower sputum viscosity by preventing NET release and chromatin decondensation. On the other hand, NE inhibitors may reduce the ability of therapeutic nucleases to promote sputum solubilization. In addition, the role of NETs in host defense against lung infections remains unclear but should not be neglected. Last but not least, we predict that small cell-permeable NE inhibitors may prove more beneficial in reducing sputum viscosity than the less permeable serpins, as they will inhibit more efficiently the release of chromatin through NET formation [3].

## Materials and Methods

### Ethics Statement and methods for CF patient sample collection

Sputum was expectorated spontaneously from 13 adult cystic fibrosis patients with proven diagnosis by sweat test and genetics, in compliance with an ethical protocol approved by the Geschäftsführung der Ethikkommission der Charité Universitätsmedizin. Anonimity was maintained for all samples collected. The patients were being treated for chronic *Pseudomonas aeruginosa* infections and none were colonized with *Bulkholderia cepacia* or multidrug resistant *Staphylococcus aureus*. Processing was initiated approximately 1 hr after donation.

### Immunostaining and microscopy

Sputum was fixed in 4% parafolmadehyde, dehydrated, embedded into paraffin and sectioned to 5 µm thin slices. Following deparaffinization, the samples were blocked with 5% BSA/5% donkey serum, and stained with rabbit anti-MPO (1∶200) (DAKO), mouse anti-NE (1∶200) (in-house) and Hoechst DNA dye, and fluorescent secondary antibodies (1∶500) (Jackson).

### Biochemical analysis of sputum

Sputum from 10 patients who received Dornase (Roche) 3–4 hrs prior to donation was separated into 3 mL aliquots and supplemented with 2 mL of PBS containing 10 mM calcium, 5 U/mL MNase (Sigma). One aliquot received no protease inhibitors, another 5 µM GW311616A (Sigma-Aldrich) (NEi), and another 5 µM SLPI (Sigma), and agitated at 37°. At the indicated time points the samples were centrifuged at 1000× g for 10 min, the soluble volume was recorded and 200 µL aliquots of the supernatant were removed and cleared at 15000× g.

### Native electrophoresis

Solubilized sputum was resolved over 1% agarose gels in 20 mM HEPES, 100 mM NaCl buffer. Gels were soaked in 5 ng/mL ethidium bromide solution for DNA detection and in a solution containing 0.1 mg/mL of O-phenylenediamine and 1 mM H_2_O_2_ to detect MPO activity. Gel contents were transferred onto PVDF membranes by semi-dry blotting.

### Western immunoblotting

Primary antibodies: anti-H3 (1∶10000), anti-H4 (1∶5000) (Upstate), anti-NE (1∶200) (Abcam ab21595), anti-MPO (1∶10000) (DAKO), anti-α1AT (1∶500) (DAKO). Secondary antibodies conjugated to horseradish peroxidase (1∶20000) (Jackson labs).

### Enzymatic assays

DNA concentrations were measured with Quant-iT PicoGreen dsDNA (Invitrogen). Protease activity measurements were performed against 300 µM elastase substrate I (Calbiochem) by monitoring absorbance at 410 nm. MPO activity in soluble samples was measured in the presence of 100 mM H_2_O_2_ by monitoring absorbance of 0.1 mg/mL of O-phenylenediamine at 450 nm.

### Cell distribution measurement

Whole sputum was diluted with equal volume of PBS containing 5 mM EGTA, 5 mM EDTA and solubilized ON at 4°C. MPO activity was measured in whole sputum aliquots and compared to the activity of low speed supernatant derived from 5×10^7^ neutrophils/mL. The activity of MPO was used to estimate the total number of neutrophils in the sputum. The number of intact neutrophils was quantified from paraffin sections stained with hematoxylin and eosin (H&E) and confirmed by immunofluorescence microscopy against NE, MPO and DNA. The neutrophils that made NETs were determined by subtracting the number of intact neutrophils from the total.

### NET quantification

The NET content of sputum in **Fig. 2b** was estimated by comparing the activity of MPO to the activity of MPO in NETs derived from isolated human neutrophils. The NET sample was prepared by plating 5×10^6^ cells and inducing with 50 nM phorbol myristate acetate for 4 hrs. DNA imaged by sytox and phase contrast images were obtained to determine the number of NETs. The culture supernatant was removed and NETs were solubilized by addition of 1 ml RPMI containing 15 u of MNase for 15 min at 37°C. The reaction was terminated by addition of 5 mM EGTA and samples were concentrated 10× using centricon YM-3 centrifugal filter units (Millipore). Sputum samples from patients receiving Dornase and supplemented with MNase were solubilized for 2 hrs at 37°C and spun at 1000 g for 10 min. Supernatant aliquots were tested for MPO activity in the presence of 100 mM H_2_O_2_ by monitoring absorbance of 0.1 mg/mL of O-phenylenediamine at 450 nm. Kinetic curves were processed with the Soft Max Pro software to obtain Vmax values. The concentration of MPO by fitting Vmax values of samples to a linear plot generated by enzymatic reactions with known concentrations of purified MPO (Calbiochem) (**Fig. S2a**).

In addition, 10 µL of each CF sample and 10 µL containing NETs derived from 4.1×10^4^ neutrophils was analyzed by native agarose gel electrophoresis, transferred on PVDF and immunoblotted for MPO. Bands were quantified from scanned film images using the ImageJ software.

## Supporting Information

Figure S1 **Sample images of CF sputum sections used in determining the intact neutrophil/NET content presented in** **Figure 1** **.** (a) Hematoxylin and eosin stain (H&E) images of paraffin sections from sputum isolated from 2 untreated patients (P1 and P2) and 2 patients receiving DNase therapy (P3 and P4). (b) Immunofluorescence images from the sputum sections in (a) stained for MPO (green) NE (red) and the DNA dye Hoechst (blue). Scale bars: 20 µm.
(TIF)

Figure S2 **Data figures used in NET quantification estimates presented in** **Fig. 1b, c** **and** **2b** **.** (a) Plot of the activity of MPO (Vmax) against the concentration of MPO. Equation of the linear fit used to calculate the amount of MPO in experimental samples. (b) Data figures used in calculating the intact cell/NET distribution in CF sputum samples (**Fig. 1b and c**). Intact cell counts were obtained from sputum sections analyzed by microscopy (**Fig. S1a and b**) The activity of MPO (Vmax) in whole CF sputum samples solubilized with EGTA was used to calculate the MPO concentration in CF sputum based on the linear fit in (a). The amount of MPO in an extract derived from 5×10^7^ neutrophils/mL was used to estimate the total number of neutrophils in sputum samples. The number of NET making neutrophils was obtained by subtracting the intact cells from the total number of cells. (c) Data figures used to estimate the NET content in CF sputum from the activity of MPO in the soluble fraction of sputum solubilized with nucleases, compared to the MPO content of NETs derived from 4.1×10^7^ neutrophils and solubilized with MNase, rather than the MPO content in whole cell neutrophil extracts (**Fig. 2b**). Vmax values of MPO activity measurements were used to calculate the MPO concentration in sputum and solubilized NET samples. The amount of MPO/NET was calculated by dividing the concentration of MPO in the “NET” control sample by the number of NETs measured by microscopy prior to solubilization with Mnase.
(TIF)
