# Supplementary figures and images for: Neutrophil Elastase Enhances Sputum Solubilization in Cystic Fibrosis Patients Receiving DNase Therapy
Source: PLoS One. 2011 Dec 9;6(12):e28526. doi: 10.1371/journal.pone.0028526 (PMC3235130; doi:10.1371/journal.pone.0028526)

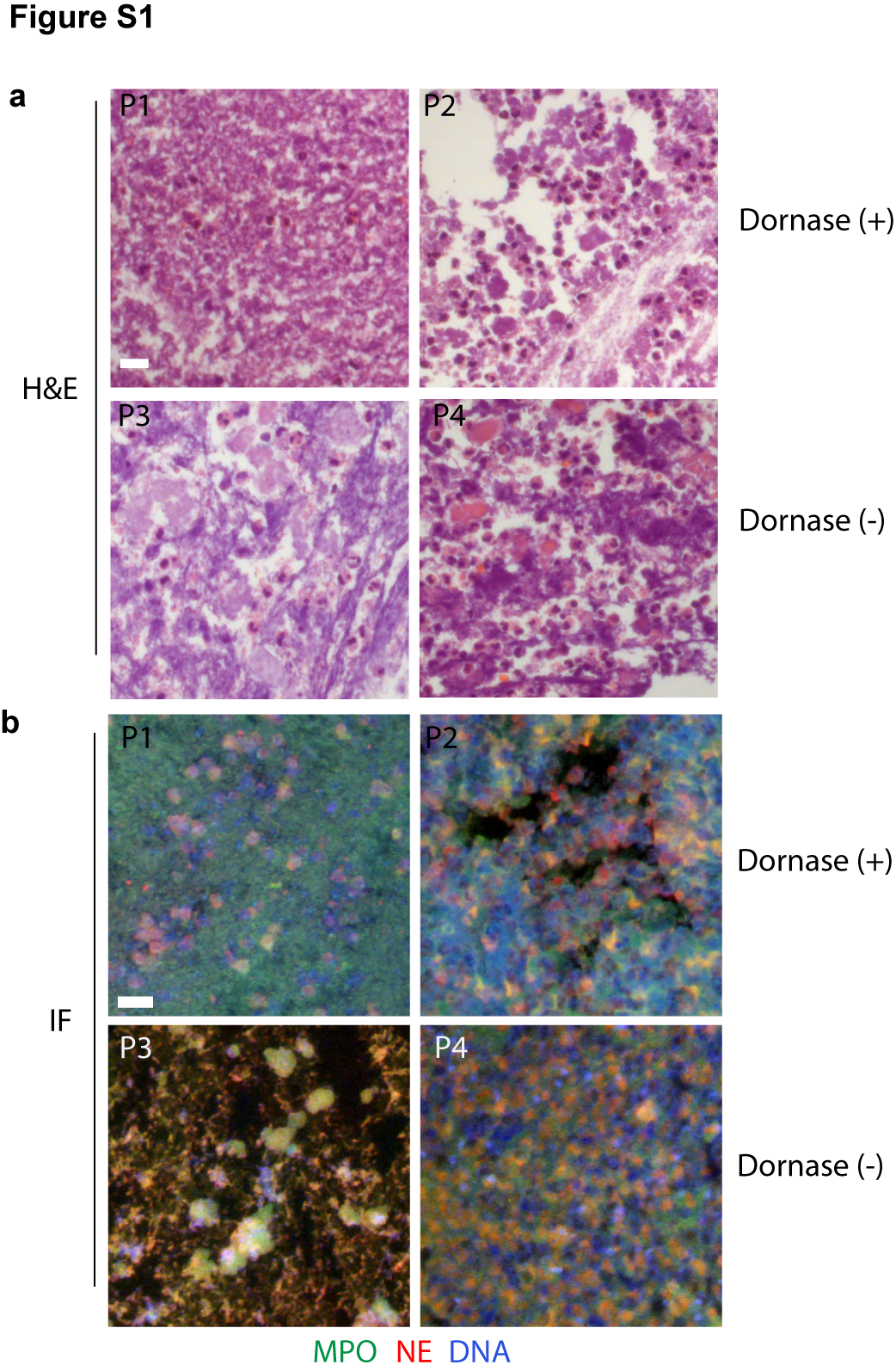

Supplement: Figure S1 — Sample images of CF sputum sections used in determining the intact neutrophil/NET content presented in Figure 1 . (a) Hematoxylin and eosin stain (H&E) images of paraffin sections from sputum isolated from 2 untreated patients (P1 and P2) and 2 patients receiving DNase therapy (P3 and P4). (b) Immunofluorescence images from the sputum sections in (a) stained for MPO (green) NE (red) and the DNA dye Hoechst (blue). Scale bars: 20 µm. (TIF) [file pone.0028526.s001.tif]

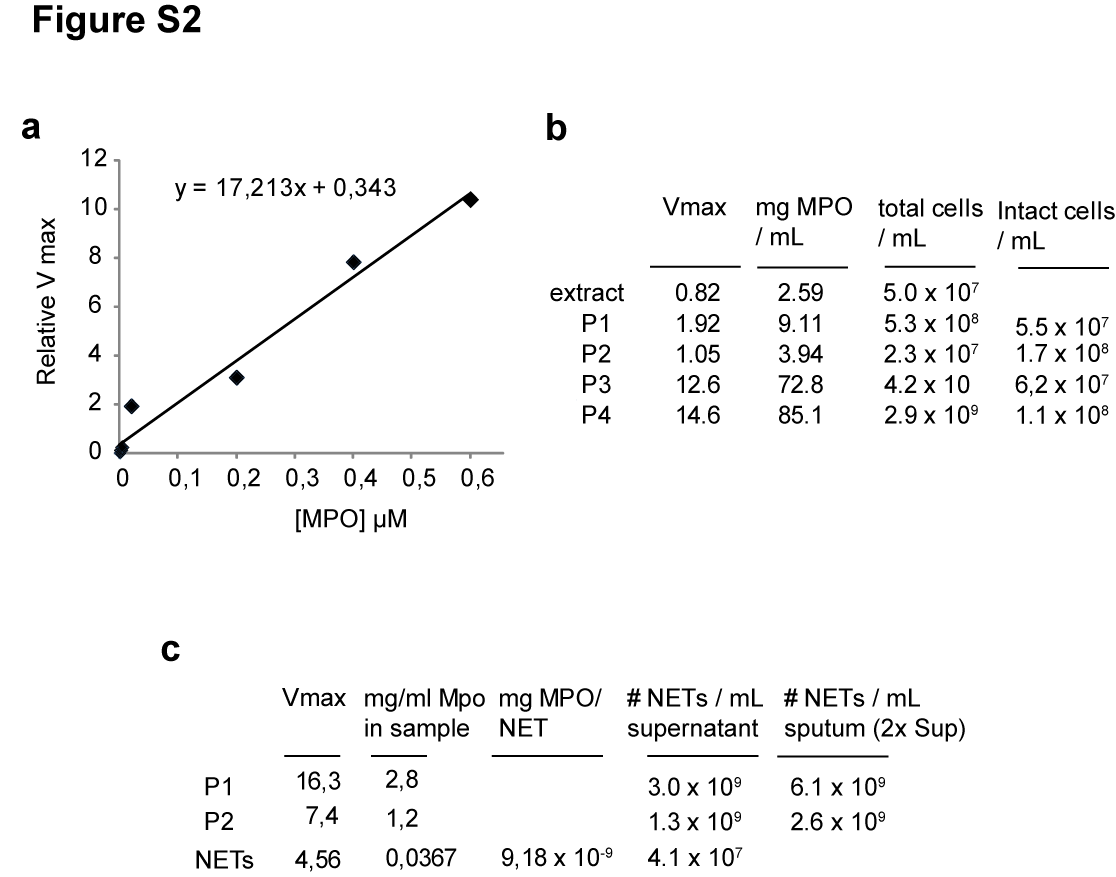

Supplement: Figure S2 — Data figures used in NET quantification estimates presented in Fig. 1b, c and 2b . (a) Plot of the activity of MPO (Vmax) against the concentration of MPO. Equation of the linear fit used to calculate the amount of MPO in experimental samples. (b) Data figures used in calculating the intact cell/NET distribution in CF sputum samples ( Fig. 1b and c ). Intact cell counts were obtained from sputum sections analyzed by microscopy (Fig. S1a and b) The activity of MPO (Vmax) in whole CF sputum samples solubilized with EGTA was used to calculate the MPO concentration in CF sputum based on the linear fit in (a). The amount of MPO in an extract derived from 5×10^7^ neutrophils/mL was used to estimate the total number of neutrophils in sputum samples. The number of NET making neutrophils was obtained by subtracting the intact cells from the total number of cells. (c) Data figures used to estimate the NET content in CF sputum from the activity of MPO in the soluble fraction of sputum solubilized with nucleases, compared to the MPO content of NETs derived from 4.1×10^7^ neutrophils and solubilized with MNase, rather than the MPO content in whole cell neutrophil extracts ( Fig. 2b ). Vmax values of MPO activity measurements were used to calculate the MPO concentration in sputum and solubilized NET samples. The amount of MPO/NET was calculated by dividing the concentration of MPO in the “NET” control sample by the number of NETs measured by microscopy prior to solubilization with Mnase. (TIF) [file pone.0028526.s002.tif]
